# Prosthodontics in Palliative Care: Optimizing Oral Rehabilitation for Quality of Life

**DOI:** 10.7759/cureus.92702

**Published:** 2025-09-19

**Authors:** Chinmoy Sikdar, Shubham K Srivastava, Akshim Rana, Shitij Srivastava, Abhinav Shekhar

**Affiliations:** 1 Department of Prosthodontics, Sardar Patel Post Graduate Institute of Dental and Medical Sciences, Lucknow, IND

**Keywords:** oral health, palliative care, prosthodontics, quality of life, rehabilitation

## Abstract

Prosthodontics plays a vital role in improving the oral health-related quality of life of patients, yet its contribution in the context of palliative care remains underexplored. Individuals receiving palliative care often suffer from oral complications such as xerostomia, mucosal ulceration, prosthesis-related trauma, and impaired mastication or speech. These conditions not only compromise nutrition and communication but also aggravate broader symptoms such as pain, dyspnea, and fatigue. For instance, poorly fitting prostheses can cause persistent oral pain, limiting food intake and thereby worsening fatigue. Difficulty in mastication and swallowing may contribute to aspiration risk, exacerbating dyspnea. By addressing these oral challenges, prosthodontic interventions can directly support comfort, reduce symptom burden, and preserve dignity in end-of-life care, complementing systemic medical management. Prosthodontic interventions, ranging from the use of soft liners and tissue conditioners to lightweight interim prostheses and modified obturators, can provide substantial relief in this setting. Despite the availability of simple and cost-effective strategies, awareness and integration of prosthodontics into palliative care pathways remain limited. This editorial highlights the urgent need to reframe prosthodontics as a discipline not confined to restorative excellence but as one that directly contributes to alleviating suffering. By emphasizing patient-centered goals, prosthodontists can enhance nutrition, communication, and psychosocial well-being for terminally ill patients. Greater collaboration between prosthodontists, oncologists, and palliative care providers is essential to ensure comprehensive oral comfort management. Addressing these gaps will not only improve patient outcomes but also align prosthodontics more closely with the core mission of palliative care, optimizing quality of life in its final stages.

## Editorial

Palliative care represents one of the most compassionate dimensions of modern health care, emphasizing comfort, dignity, and holistic well-being rather than curative intent [1]. Its primary mission is to alleviate suffering and optimize quality of life for patients facing advanced, life-limiting illnesses. Within this paradigm, symptoms such as pain, dyspnea, and fatigue often take precedence, while oral health, although integral to basic human function, is frequently overlooked [2]. Yet the mouth is central not only to nutrition and speech but also to identity, emotional expression, and social interaction. Impairment in oral function, therefore, resonates at multiple levels, affecting physical comfort, psychosocial stability, and interpersonal communication [3,4]. The neglect of oral rehabilitation in palliative pathways is thus a paradox: while systemic symptoms receive constant clinical attention, oral suffering remains inadequately addressed, despite robust evidence that targeted prosthodontic interventions can transform the lived experience of terminally ill patients [1-5].

The magnitude of the problem is substantial. Oral complications in palliative populations are not occasional inconveniences but routine clinical realities. Silva et al. conducted a comprehensive systematic review and meta-analysis across a large cohort and reported strikingly high prevalence rates: dental caries affected nearly one-third of patients, oral candidiasis was present in approximately 17%, and xerostomia and mucosal discomfort were pervasive across multiple cohorts [1]. These findings highlight a previously underappreciated burden that is often overlooked in palliative care settings. The implications are profound: patients who cannot chew comfortably face nutritional deficits; those with ulcerated mucosa endure constant pain; and those who are edentulous or struggling with prosthesis-related trauma may lose the ability to communicate clearly at the very stage of life when meaningful interaction with loved ones becomes most essential. Oral complications are not isolated discomforts; they are determinants of dignity [1,2].

Recent prospective evidence highlights the magnitude of these issues. Chen et al. conducted a large mixed-method study and found that 83.7% of patients in palliative settings reported persistent dry mouth, 51% described functional limitations in eating or speaking, and over 40% reported active oral pain [2]. An important finding was that many patients did not initially prioritize these symptoms, adapting silently until discomfort became intolerable. This underscores two critical points: first, that oral health is indeed a significant concern in advanced illness; and second, that its underreporting is partially a consequence of both patient adaptation and clinical neglect. If patients internalize the idea that oral suffering is unavoidable, and if clinicians seldom inquire proactively, a vicious cycle of neglect ensues. Breaking this cycle requires active involvement of dental professionals, particularly prosthodontists, whose expertise extends beyond disease control to functional rehabilitation. Consistent oral care helps maintain oral hygiene, lowers the incidence of mucositis, alleviates dryness by improving moisture levels, and decreases the risk of infections in patients nearing the end of life. Providing daily oral care not only relieves discomfort but also supports better food intake and facilitates communication between terminally ill patients and their families [6]. Matsuo et al. reported that oral complications in palliative care tend to occur more frequently when the days-to-death (DTD) period is shorter [7]. Such manifestations may serve as valuable indicators for determining the appropriate timing of intensive oral care interventions aimed at reducing oral discomfort and pain in terminally ill patients.

Encouragingly, intervention studies provide consistent evidence that even modest prosthodontic or oral care measures can yield tangible improvements. Dhaliwal et al. conducted a systematic review of interventional approaches and found that measures such as denture relining with soft tissue conditioners, occlusal adjustments, fabrication of lightweight interim dentures, and simplified obturators for maxillofacial defects directly reduced pain and enhanced oral function in palliative patients. However, these strategies are not without challenges; soft liners may degrade over time and harbor infection, prosthesis durability can be limited in fragile patients, and adherence to maintenance protocols is often inconsistent due to reduced compliance in end-of-life settings [3]. These interventions diverge from traditional prosthodontic practice, which often prioritizes durability and esthetics, by instead emphasizing immediate comfort, minimal invasiveness, and functional adequacy. For example, a frail patient with advanced cancer may not tolerate the complexity of definitive prosthesis fabrication, but can benefit immensely from a soft relining material that cushions sore mucosa or from a lightweight removable denture that restores minimal chewing ability. Such adjustments, though simple, restore a sense of normalcy in eating, speaking, and social interaction, thereby aligning prosthodontics with the true ethos of palliative care.

Serra et al. further highlighted the importance of structured oral hygiene practices. Their mixed-methods systematic review emphasized that consistent oral hygiene interventions, ranging from basic cleaning protocols to saliva substitutes, mitigated xerostomia and mucositis in palliative cancer patients [4]. Importantly, they observed that where oral hygiene protocols were routinely applied, improvements in well-being were evident, yet in many care settings these measures were inconsistently implemented. For prosthodontists, this carries two implications: first, that oral rehabilitation is not limited to device fabrication but also includes the reinforcement of preventive and hygiene protocols; and second, that integration of such measures into daily care is essential for sustained comfort. A prosthesis that restores function but is poorly maintained in a xerostomic, immunocompromised patient may quickly become a nidus of infection. Thus, rehabilitation and hygiene must go hand in hand.

The integration of prosthodontics within palliative teams has also been shown to confer broader benefits. Uhlig et al. reported on a monocentric study involving 103 palliative inpatients and found that bedside dental interventions, including prosthesis adjustments and oral hygiene measures, significantly reduced sticky saliva, mucosal soreness, and denture-related problems, translating into measurable improvements in oral health-related quality of life [5]. What is particularly noteworthy is that these improvements were not only functional but also psychosocial. Patients who regained the ability to speak without embarrassment or eat without pain demonstrated enhanced emotional well-being and greater engagement with caregivers and family members. This underscores that prosthodontic rehabilitation extends beyond technical repair; it enables meaningful communication, restores social confidence, and protects dignity in the final stage of life.

Despite the growing body of evidence, dentistry and prosthodontics in particular remains poorly integrated into global palliative care frameworks. Many hospitals and oncology centers do not employ dental professionals, and referrals are often reactive rather than proactive. This systemic gap reflects a broader cultural undervaluation of oral health in terminal care. The ethical dimension is unequivocal: if the central mission of palliative care is to relieve suffering in all its forms, then neglecting oral rehabilitation directly undermines this mandate. Although the WHO's public health model for palliative care and various national frameworks emphasize pain, nutrition, and communication as essential domains, oral health remains largely absent from these standards. This omission underscores that oral comfort and function are not merely clinical issues but fundamental human rights, intrinsically linked to dignity, nutrition, communication, and self-expression. Oral comfort and function are human rights issues, directly linked to dignity, nutrition, communication, and self-expression. As Serra et al. noted, low-cost, protocol-driven oral care interventions already exist and have proven efficacy, meaning that barriers to implementation are not primarily financial or technical but attitudinal and systemic [4].

Educational deficits also perpetuate the problem. Most medical and nursing curricula offer little to no training on oral health in palliative contexts, leaving frontline clinicians ill-equipped to identify or manage prosthesis-related trauma, candidiasis, or xerostomia. Researchers emphasized that oral complications remain underreported partly because they are underrecognized by clinicians [1,4]. Without basic awareness, opportunities for timely prosthodontic intervention are missed. Reform is therefore essential: curricula must integrate oral health into palliative education, guidelines must standardize oral care practices, and prosthodontists must be included systematically in interdisciplinary teams. Only by bridging this knowledge gap can oral rehabilitation assume its rightful place in holistic care. Survey data from dental and dental-hygienist training programs in Japan show that palliative care content is minimal, especially regarding oral symptoms, end-of-life physical changes, and interdisciplinary roles [8].

Looking toward the future, innovations in prosthodontic materials and techniques hold promise for enhancing palliative outcomes. Moisture-retaining prostheses for xerostomic patients, antimicrobial soft liners to reduce mucosal infections, and digitally fabricated lightweight dentures that minimize strain are examples of advances tailored to the needs of fragile populations. Additive manufacturing technologies and chairside digital workflows may soon enable rapid production of customized interim prostheses, reducing treatment burden while maximizing benefit. However, the adoption of these technologies in low-resource palliative settings remains limited by cost, infrastructure, and training constraints. Bridging this feasibility gap will require phased implementation strategies, context-appropriate adaptations, and policy support to ensure equitable access. Dhaliwal et al. demonstrated that even existing interventions are effective; the challenge now is to adapt emerging technologies to the unique priorities of palliative care [3]. Here, research should not focus exclusively on longevity or esthetics but on comfort, adaptability, and patient-reported outcomes.

The broader lesson from this evidence is clear: prosthodontics in palliative care is not an adjunct luxury but an ethical necessity. Oral rehabilitation alleviates pain, restores function, enhances communication, and safeguards dignity. The literature consistently documents the prevalence of oral complications [1,2], the effectiveness of targeted interventions [3,4], and the improvements possible through team integration [5]. Together, these findings form a compelling case for systemic change. Medical curricula must incorporate oral health, palliative policies must mandate access to dental expertise, and interdisciplinary teams must include prosthodontists as standard members. Without these changes, patients will continue to endure preventable suffering.

In conclusion, the role of prosthodontics in palliative care must be reframed. It is not merely a discipline concerned with restoring lost structures for esthetics or long-term stability, but one dedicated to optimizing oral rehabilitation as a means of alleviating suffering and enhancing quality of life in the final stages of illness. Evidence demonstrates that relatively simple interventions, such as soft liners, interim dentures, obturators, and structured hygiene protocols, can profoundly improve comfort and psychosocial well-being. Emerging materials and technologies offer new avenues for tailoring care to the fragile oral environment of palliative patients. Yet the most urgent task is systemic: integrating prosthodontics into palliative care as a standard practice, supported by education, policy, and interdisciplinary collaboration. To deny oral rehabilitation is to deny dignity, and to integrate it is to honor the very essence of palliative medicine. The challenge before us is not one of evidence but of will. By embracing this responsibility, prosthodontists can help ensure that patients at the end of life retain not only the ability to eat, speak, and smile but also the comfort, dignity, and human connection that define quality of life itself.
